# Response of some faba bean genotypes to heat stress under environmental variations

**DOI:** 10.1186/s12870-026-09316-w

**Published:** 2026-06-22

**Authors:** Mohamed R. Asaad, Essam F. El-Hashash, Ahmed R. M. Ridwan

**Affiliations:** 1 Agronomy Department, Faculty of Agriculture, Minia University, Minya, 61519 Egypt; 2 Agronomy Department, Faculty of Agriculture, Al-Azhar University, Nasr City, Cairo 11651 Egypt; 3 Agronomy Department, Faculty of Agriculture, Sohag University, Sohag, 82524 Egypt

**Keywords:** Environments, Genotypes, Stress indices, Univariate models, Multivariate models, Faba bean

## Abstract

The faba bean productivity is affected by high temperatures during the growing season. Therefore, faba bean genotypes must be developed to tolerate heat stress. In this study, nine genotypes were assessed over two years and two locations under normal and heat-stress conditions to identify high-yielding, and heat-tolerant genotypes. Seed and straw yields, along with most of the traits under study, showed high genotype diversity and genetic variability across years and locations under in both conditions, according to the combined ANOVA. Seed yield and other traits under investigation were significantly reduced by heat stress, yielding values lower than under normal conditions. When comparing the two years and two locations, the 2023/2024 growth year and the El-Minya location under both conditions had the best values for the majority of the attributes under study. Nubaria 3, Giza 843, and Giza 429 genotypes showed the capacity to produce earlier and increase seed yield/plant, and were among the most investigated attributes under heat-stress conditions, according to the mean performance. MP, GMP, STI, and HM stress indices were associated with both normal and heat-stress conditions; thus, they were more effective at identifying high-yielding genotypes. The genotypes Giza 843, Nubaria 3, and Giza 429 exhibit heat tolerance as indicated by stress tolerance indices. A strong positive correlation was found among SY/P, BY/P, NP/P, and SP traits, as well as among other studied traits in both conditions. The PCs biplot effectively separated genotypes associated with higher seed yield and related traits from those associated with lower grain yield and related traits. These traits, based on univariate and multivariate models, can be used for direct selection to increase faba bean productivity under heat stress. According to statistical analyses, the Nubaria 3, Giza 843, and Giza 429 genotypes have significant potential under heat-stress conditions. Therefore, they could serve as a source of short-duration cultivars to enhance seed and straw yields in the Egyptian faba bean breeding program in the future.

## Introduction

According to the IPCC, heat wave periods of unusually hot weather lasting days to weeks are predicted to become more intense and frequent due to climate change, and the average global temperature is also predicted to rise by 1.5 °C [1]. Crop production will be severely impacted in many areas, particularly in the Mediterranean region and in Nile Valley nations such as Sudan and Egypt, where faba beans are widely grown as a key food legume [2]. The degree of high temperature, duration of exposure, and intensity all affect the effects of heat stress [3].

Among legumes, the faba bean (*Vicia faba* L.) is a significant protein crop used for food and feed worldwide. It offers several benefits for crop rotation. The primary reason for its limited application in contemporary agriculture, which is the substantial variability in production [4]. This emphasizes the pressing need to increase faba bean production in the area, which calls for modifying agronomic practices and creating more productive, locally adapted cultivars that are resistant to the primary biotic stressors in the cultivated regions of the Mediterranean basin, as well as heat and drought-tolerant [5]. Heat stress is often the most destructive of the various obstacles to faba bean production, particularly in the Mediterranean and Nile Valley regions where faba beans are farmed as an essential legume crop [6]. The growth and productivity of faba beans are severely limited by heat stress. Faba bean plants’ physiology is affected by heat stress in several ways, including reduced chlorophyll variable fluorescence, growth rate, membrane stability, photosynthetic pigment concentrations, and particularly sensitive reproductive processes [7]. Pollen viability is compromised by temperatures above 30 °C during the blooming stage, thereby reducing pod set and significantly lowering yield [7, 8]. Breeding for heat tolerance is therefore becoming increasingly important in light of the documented rise in temperatures [6].

Seed yield is a complex ex trait that is directly or indirectly related to other morphological and physiological traits and is strongly influenced by the environment [9]. Faba bean production is greatly affected by soil and climate variability, especially in light of current climate changes [10], with high heat stress being a particular concern. Breeders and agronomists can identify and understand genotype-by-environment interaction for the best-performing genotypes and rank genotypes across diverse environments by studying cultivars in diverse environments [11]. To identify and recommend stable, widely adapted genotypes for production in the target environments, it is crucial to conduct experiments across years and locations to aid our understanding and analysis of the genotype-by-environment interaction [12]. When evaluating new top-yielding cultivars under heat-stress, years and location trials are helpful. Developing varieties that are appropriate for the areas in which faba beans are grown is the most efficient way to overcome biotic and abiotic limits on faba bean yield [13].

Selection under favorable conditions is supported by some researchers [14], whereas selection under stress conditions is supported by others [15]. However, many researchers believe in selection under both favorable and stressful conditions and choose the middle ground [16, 17]. Based on a mathematical link between yield under stress and non-stressed conditions, several stress indices have been proposed to distinguish stress-tolerant genotypes. These indices are based on genotypes that are either susceptible to or resistant to stress [18]. Therefore, the performance consistency of the genotypes under stress is confirmed by the use of multiple stress indices [19]. To select stress-tolerant genotypes with good yield performance under both stress and non-stress conditions, various stress indicators have been consistently associated with other indices [4]. Researchers studying faba beans have therefore used a variety of stress tolerance indices to assess crop responses to environmental stresses and to screen for genotypes that are susceptible and tolerant [4, 5, 19–22].

The aforementioned makes it clear that faba bean yield stability under various soil and climatic conditions is a crucial component of a sustainable farming system, particularly given the significant year-to-year variations in climatic conditions today. The current study used various statistical analyses to: (1) evaluate nine genotypes of faba beans in two years and two locations under normal and heat stress conditions; (2) identify genotypes with the highest grain yield and earliness that have the potential for both specific and wide adaptation under heat stress conditions; and (3) identify a location that is more suitable for faba bean cultivation under heat stress conditions in Egypt. Seed and straw yields, along with other traits studied, were evaluated to test our hypothesis that high temperatures negatively affect faba bean production and to identify tolerant genotypes.

## Materials and methods

### Experimental site and climate-soil conditions

Field experiments were conducted at two sites: the experimental farms of the Faculty of Agriculture at Minia University in El-Minya Governorate, Middle Egypt (latitude 28°07’03.1” N and longitude 30°43’51.8” E) and the Agricultural Research Farm at Al-Kawamel site, Faculty of Agriculture, Sohag University, Sohag Governorate, Upper Egypt (26°27’38.4"N 31°40’01.9"E), during the consecutive winter seasons of 2023/2024 and 2024/2025. Two trials were conducted at each location in each season, with sowing dates of November 1st (heat-stress conditions) and December 1st (normal conditions). The soil physical and chemical properties and soil moisture constants at the two experimental sites, determined according to Jackson [23], are listed in Table 1.

**Table 1:** Soil physical and chemical properties at the El-Minya and Sohag (means of 2023/2024 and 2024/2025 seasons)

Soil Characteristics	Locations	
	El-Minya	Sohag
Texture	Clay	Sandy Loam
Sand (%)	17.76	67
Silt (%)	25.79	18
Clay (%)	56.45	15
pH*	7.70	8.2
EC (dS. m-1) (1:5)	1.35	1.45
Ca CO3 (%)	1.79	2.55
Total N (%)	0.13	0.09
Available P (ppm)	13.11	13.71
Available K (ppm)	437.0	155.7
Organic Matter (%)	2.86	0.27

*pH was determined in a 1:2.5 (w: v) water suspension for El-Minya soil and 1:1 (w: v) for Sohag soil

The minimum, maximum, and mean daily temperature as well as relative humidity at monthly intervals during the 2023/2024 and 2024/2025 growing seasons, are shown in Table 2. There were notable differences between the two years and between the two study locations due to differences in mean temperature and average relative humidity. The 2023/2024 experimental year and the Sohag location showed higher temperatures from sowing to harvest, resulting in more intensive heat stress than the 2024/205 year and the El-Minya location, respectively. The highest heat was observed in October and April months during both years and locations. Also, there was greater heat stress in November than in December in both years and locations. Additionally, compared to other years and locations under investigation, we observed higher average relative humidity at the El-Minya location and during the 2023/2024 growing seasons. In both years and locales, December and April had the highest and lowest average relative humidity, respectively.

**Table 2:** Monthly meteorological data for El-Minya and Sohag locations during 2023/2024 and 2024/2025 growing seasons

Locations	Months	2023/2024 growing season				2024/2025 growing season			
		Air temperature °C			RH%	Air temperature °C			RH%
		Min	Max	Avg.		Min	Max	Avg.	
El-Minya	Oct.	15.86	34.36	24.85	47.24	12.81	37.21	23.93	45.58
	Nov.	8.80	32.14	19.53	55.53	7.09	32.06	18.04	55.14
	Dec.	7.53	26.32	15.51	64.44	3.32	26.46	13.39	59.06
	Jan.	3.31	25.95	12.46	54.09	5.34	24.85	14.18	58.08
	Feb.	2.86	27.70	13.38	53.86	3.17	23.26	12.36	53.99
	Mar.	6.67	34.15	17.74	42.96	5.78	35.94	18.47	41.59
	Apr.	11.41	40.96	23.6	33.52	8.94	38.91	22.63	32.17
Sohag	Oct.	16.74	38.30	27.11	35.62	13.25	38.62	25.72	32.81
	Nov.	10.28	36.90	22.81	40.15	7.27	32.02	19.16	45.66
	Dec.	7.51	31.39	17.31	52.84	4.39	29.68	14.79	46.89
	Jan.	2.95	28.73	14.01	46.62	6.25	31.34	16.24	45.34
	Feb.	2.64	29.79	14.64	45.59	3.97	28.72	14.12	40.35
	Mar.	8.52	35.91	20.39	28.85	7.53	38.15	20.98	29.66
	Apr.	13.32	42.43	26.27	23.25	11.13	44.51	25.45	22.11

### Genetic material and field procedure

In each experiment, nine faba bean (*Vicia faba* L.) varieties (Table 3) with diverse genetic backgrounds and varying performance traits, were used. These genotypes were Giza 843, Giza 716, Giza 429, Wadi 1, Sakha 1, Nubaria 3, Nubaria 1 and Misr 3, sourced from the Field Crops Research Institute (FCRI) at the Agricultural Research Center (ARC), Egypt. At the same time, Mariout 2 was obtained from the Desert Research Center (DRC). For each sowing date (normal and stress conditions) across both years and locations, the experiments were laid out in a Randomized Complete Block Design (RCBD) with three replications. The experimental plot area was 9.6 m², comprising four ridges, each 4 m long and 0.6 m wide. Sowing was performed using the hand-dry planting method on one side of the ridge. Seeds were placed in hills spaced 20 cm apart, with two seeds per hill to ensure optimum plant density. Regarding fertilization, phosphorus was applied during soil preparation at a rate of 20 kg P₂O₅ fed⁻¹ in the form of calcium superphosphate (15.5% P₂O₅). Additionally, nitrogen fertilization was provided at a rate of 20 kg N fed⁻¹ using urea (46.0% N). All other agronomic practices were applied in accordance with standard recommendations for faba bean production in the El-Minya and Sohag regions.

**Table 3:** Pedigree of the studied faba bean genotypes used in this study

No.	Genotype	Pedigree
1	Giza 843	561/2076/85 Sakha x461/845/83
2	Giza 716	Crossing between (416/842/83 × 503/453/83)
3	Giza 429	Individual plant selection from Giza 402
4	Wadi 1	Rena Blanka × Triple white
5	Sakha 1	Giza 716 × 620/ 283/ 85
6	Nubaria 3	Selection in Ahnasiaz
7	Nubaria 1	Individual plant selection Spanish variety Rena Blanka
8	Misr 3	667x (Cairo 241x Giza 461)
9	Mariot 2	(2095/76 × ILB 1550)

### Traits measurement

Days from sowing to 50% flowering (DSF; day) and days from sowing to 90% maturity (DSM; day) were recorded. At harvest, plant height (PH, cm), the number of branches/plant (NB/P), the number of pods/plant (NP/P), 100-seed weight (100-SW; g), seed yield/plant (SY/P; g), straw/plant (S/P; g), Biological yield/plant (BY/P; g), and harvest index (HI; %) were recorded using a random sample of 10 guarded plants from the central ridges of each plot.

### Statistical analysis

A combined analysis of variance was performed to determine the effects of years, locations, genotypes and their interactions on phenotypic data under normal and heat-stress conditions, according to the method of Gomez and Gomez [24]. The coefficient of variation (C.V.%) was estimated and categorized as very high (C.V.% ≥ 21), high (15 ≤ C.V.% < 21), moderate (10 ≤ C.V.% < 15), and low (C.V.% < 10) according to Pimentel-Gomes [25]. To distinguish between genotypes based on heat-stress response, stress tolerance indices for faba bean seed yield/plant under normal (Yp) and heat-stress (Ys) conditions were computed for each variety using the methods listed in Table 4. To better understand the relationships among the experimental factors, stress tolerance indices, and the traits under study, principal component analysis (PCA) and cluster analysis were employed. The ANOVA and multivariate analysis were performed using SPSS version 20 and OriginPro 2025b 10.2.5.212, respectively.

**Table 4:** Heat tolerance indices were used to evaluate nine faba bean genotypes under heat-stress conditions

No.	Heat tolerance indices	Equation	Reference
1	Stress susceptibility index (SSI)	$\:[1-({Y}_{s}/{Y}_{p}\left)\right]/[1-({\stackrel{-}{Y}}_{s}/{\stackrel{-}{Y}}_{p}\left)\right]$	Fischer and Maurer [26]
2	Stress tolerance index (TOL)	$\:{\mathrm{Y}}_{\mathrm{p}}-{\mathrm{Y}}_{\mathrm{s}}$	Rosielle and Hamblin [27]
3	Mean productivity index (MP)	$\:{(Y}_{p}+{Y}_{s})/2$	Rosielle and Hamblin [27]
4	Geometric mean productivity (GMP)	$\:{\left({\mathrm{Y}}_{\mathrm{p}}{\mathrm{x}\mathrm{Y}}_{\mathrm{s}}\right)}^{1/2}$	Fernandez [17]
5	Stress tolerance index (STI)	$\:{(Y}_{p}x{Y}_{s})/{\left({\stackrel{-}{Y}}_{p}\right)}^{2}$	Fernandez [17]
6	Yield index (YI)	$\:{Y}_{s}/{\stackrel{-}{Y}}_{s}$	Gavuzzi et al. [28]
7	Yield stability index (YSI)	$\:{Y}_{s}/{Y}_{p}$	Bouslama and Schapaugh [29]
8	Stress resistance Index (SI)	$\:{[Y}_{s}x({Y}_{s}/{Y}_{p})]/{\stackrel{-}{Y}}_{s}$	Lan [30]
9	Yield reduction ratio (YR)	$\:1-{(Y}_{S}/{Y}_{p})$	Golestani–Araghi and Assad [31]
10	Abiotic tolerance index (ATI)	$\:\left[{(Y}_{p}-{Y}_{s})/({\stackrel{-}{Y}}_{p}-{\stackrel{-}{Y}}_{s})\right]x\left[\sqrt{{Y}_{p}x{Y}_{s}}\right]$	Moosavi et al. [32]
11	Stress susceptibility percentage index (SSPI)	$\:\left[{(Y}_{p}-{Y}_{s})/2({\stackrel{-}{Y}}_{p})\right]x100$	Moosavi et al. [32]
12	Harmonic mean (HM)	$\:\left[2\left({Y}_{p}x{Y}_{s}\right)\right]/({Y}_{p}+{Y}_{s})$	Hossain et al. [33]
13	Golden mean (GOL)	$\:{(Y}_{p}+{Y}_{s})/{(Y}_{p}-{Y}_{s})$	Moradi et al. [34]

$$Y_p\;\mathrm{and}\;Y_s\;;\;\mathrm{seed}\;\mathrm{yield}\;\mathrm{of}\;\mathrm{each}\;\mathrm{genotype}\;\mathrm{under}\;\mathrm{normal}\;\mathrm{and}\;\mathrm{heat}\;\mathrm{stress}\;\mathrm{conditions},\;\mathrm{respectively}$$

$$\overline{Y_p}\;\mathrm{and}\;\overline{Y_s}\;;\;\mathrm{mean}\;\mathrm{seed}\;\mathrm{yield}\;\mathrm{of}\;\mathrm{all}\;\mathrm{genotypes}\;\mathrm{in}\;\mathrm{normal}\;\mathrm{and}\;\mathrm{heat}\;\mathrm{stress}\;\mathrm{conditions},\;\mathrm{respectively}$$

## Results and discussions

### Analysis of variance

The differences between nine faba bean genotypes over two years and two locations under normal and heat-stress circumstances were confirmed by independent analysis of the data using the statistical model, as shown in Table 5. The combined analysis of variance showed that all studied traits were significantly affected by genotypes (G) at the 1% probability level in both conditions. These findings demonstrate significant diversity among genotypes [35] and genetic variability in the traits [3]. While the years (Y) had a significant (*P* < 0.05 or 0.01) impact on all examined traits in both conditions, except for 90% maturity and number of branches/plant in normal conditions, and for number of pods/plant in heat stress conditions. Due to fluctuations in meteorological conditions of the growing season, faba bean properties vary significantly from year to year. As for locations (L), highly significant differences were found for all studied traits, except for plant height under normal conditions. The results of the ANOVA are in line with research by Al-Fartossi and Alrubaiee [36], Chetto et al. [35], El-Abssi et al. [37], Fenn et al. [38], Katsoulieri et al. [39], and Sallam et al. [40], who also confirmed a significant effect on the majority of faba bean traits in relation to the year, location, and genotype. For each variable examined under heat stress, significant differences in locations and genotype were also noted [2]. Earliness, plant height, seed yield, and yield component traits are all significantly influenced by the environment, reflecting differences in soil-climatic conditions across sites and growing season [12].

**Table 5:** Combined ANOVA of two years, two locations and nine genotypes for studied traits in faba bean under normal (N) and heat stress (S) conditions

S.O.V	df	DSF		DSM		PH		NB/*P*		NP/*P*	
		*N*	S	*N*	S	*N*	S	*N*	S	*N*	S
Year (Y)	1	9.36^**^	181.02^**^	1.66^ns^	268.64^**^	517.99^**^	677.82^**^	1.57^ns^	1.75^**^	14.88^*^	25.25^ns^
Locations (L)	1	7675.07^**^	3085.06^**^	12289.14^**^	57.35^**^	8.76^ns^	1983.16^**^	8.77^**^	0.56^**^	1410.59^**^	2283.62^**^
Y x L	1	0.31^ns^	70.34^**^	8.37^*^	74.45^**^	26.94^*^	25.33^ns^	0.09^ns^	0.88^**^	42.93^**^	28.81^ns^
Replications(YxL)	8	0.63^ns^	0.80^ns^	2.01^ns^	5.18^*^	5.57^ns^	8.75^ns^	0.27^ns^	0.07^ns^	3.64^ns^	13.14^**^
Genotypes (G)	8	28.14^**^	16.78^**^	156.77^**^	54.06^**^	157.08^**^	163.07^**^	2.74^**^	1.63^**^	29.23^**^	24.53^**^
Y x G	8	2.56^**^	4.90^**^	14.35^**^	18.83^*^	8.81^ns^	20.94^*^	0.76^**^	0.48^**^	13.95^**^	1.75^ns^
L x G	8	24.32^**^	16.93^**^	73.35^**^	39.27^**^	40.19^**^	79.66^**^	0.78^**^	0.33^*^	36.40^**^	28.15^**^
Y x L x G	8	1.99^**^	3.11^**^	9.84^**^	15.75^**^	8.66^ns^	19.92^*^	0.43^ns^	0.36^**^	11.89^**^	4.62^ns^
Error	64	0.36	0.88	1.90	2.35	9.00	7.78	0.19	0.13	3.18	3.35
CV%		1.25	2.02	0.86	1.02	2.79	2.74	10.79	10.31	11.64	14.44

S.O.V	df	100-SW		SY/P		S/P		BY/P		HI	
		N	S	N	S	N	S	N	S	N	S
Year (Y)	1	203.29^**^	1218.69^**^	775.54^**^	133.59^*^	11639.72^**^	106.86^**^	18424.26^**^	479.41^**^	263.38^**^	51.99^**^
Locations (L)	1	8510.47^**^	1757.67^**^	2153.92^**^	4128.28^**^	32164.36^**^	27278.68^**^	50965.11^**^	52630.92^**^	1596.50^**^	640.89^**^
Y x L	1	52.23^*^	591.30^**^	1523.78^**^	56.76^ns^	14156.32^**^	24.58^**^	24969.04^**^	156.04^**^	124.11^**^	32.54^**^
Replications(YxL)	8	11.42^ns^	5.22^ns^	22.34^**^	19.60^**^	77.96^*^	2.77^ns^	167.20^**^	17.31^ns^	3.04^ns^	13.80^**^
Genotypes (G)	8	48.07^**^	41.24^**^	109.81^**^	59.15^**^	145.16^**^	141.84^**^	287.06^**^	318.65^**^	58.78*^*^	27.32^**^
Y x G	8	61.36^**^	77.01^**^	21.95^**^	15.85^*^	219.95^**^	38.63^ns^	347.16^**^	34.54^ns^	7.67^ns^	23.49^**^
L x G	8	55.13**	82.47^**^	39.43^**^	112.77^**^	233.52^**^	26.99^ns^	393.43^**^	228.36^**^	24.17^**^	47.35^**^
Y x L x G	8	47.04^**^	77.29^**^	28.92^**^	10.53^ns^	201.67^**^	60.23^*^	278.04^**^	42.96^ns^	18.86^**^	25.41^**^
Error	64	6.53	4.33	7.66	6.65	30.89	21.71	54.64	40.17	3.99	4.17
CV%		3.34	2.71	9.33	11.08	10.29	10.50	8.38	9.37	5.39	5.80

*df* degree of freedom, *CV*% Coefficient of variation, * and ** Itatistically significant differences at *p* ≤ 0.05 and *p* ≤ 0.01 Respectively, *ns* Insignificant difference, *DSF* Days from sowing to 50% flowering, *DSM* Days from sowing to 90% maturity, *PH* Plant height, *NB/P* The number of branches/plant, *NP/P* The number of pods/plant, *100-DW* 100-seed weight, *SY/P* Seed yield/plant, *S/P* Straw/plat, *BY/P* Biological yield/plant, *HI* Harvest index

The first-order interaction effects (YL, YG, and LG) were significant (*p* < 0.05 or 0.01) for most traits evaluated in both conditions. The second-order interaction (YLG) had a significant effect at the 0.05 and 0.01 probability levels for all studied traits, except for plant height and number of branches/plant traits under normal conditions, and for number of pods/plant and biological yield/plant traits under heat stress conditions. The genotype x location interactions [39, 40], genotype x environment interaction [2, 5, 12, 37], and interactions of variety, growing location, and year [38] demonstrated significant or highly significant differences across all traits examined. According to Falconer and Mackay [41], the existence of first- and second-order interactions suggests that a genotype’s phenotypic expression may be superior to another genotype in one environment but inferior in another. The interaction between genotype and environment is known to be significantly reduced by genotype choices that interact less with the environment in which they are to be grown [42]. The significant GEI interaction discovered reveals disparities in performance across the environments under investigation and highlights their distinct discriminative capacities.

The values of the experimental coefficient of variation (CV%) were lower than 10% for all studied traits, except the number of branches/plant, number of pods/plant, and straw yield/plant traits in both conditions, and for seed yield/plant across heat stress conditions (10% < CV < 15%). These results showed that the environmental influence was low for all studied traits and moderate for the four previously studied traits. The CV% values indicated that the genotypes had exploitable genetic variability for the variables under investigation. For every trait under study, the environments (years and locales) accounted for a significant portion of the overall variation, as reported by El-Abssi et al. [37], Fenn et al. [38], Papastylianou et al. [12], and Temesgen et al. [11]. Higher mean squares values attributed to environments indicate significant variations between environments for all traits under investigation. These findings show that the genotypes under study exhibited high genetic variability (diversity) across two years and two sites, which could be utilized in a faba bean breeding program. The broad ranges observed for each attribute are also reflected here. Furthermore, there was sufficient genetic variety to enable the selection of genotypes to raise Egypt’s faba bean productivity.

### Mean performances

Table 6 lists the primary effects of years, locations, and faba bean genotypes for all investigated traits. Compared between the two years, the highest values for all studied traits were recorded in the 2023/2024 growing year in both conditions, whereas 50% flowering and 90% maturity traits recorded the minimum values (desirable) during the 2024/2025 growing year in both conditions. As for the locations, the El-Minya location registered the highest values of number of pods/plant, seed yield/plant, straw yield/plant, and biological yield/plant traits in both conditions and plant height in stress conditions, as well as the minimum values of 50% flowering in both conditions and of 90% maturity in normal conditions (desirable). While the maximum number of branches/plants, 100 seeds weight, and harvest index traits in both conditions, and plant height in normal conditions, as well as the minimum 90% maturity in stress conditions, were observed at the Sohag location (desirable). Significantly higher seed and straw yields/plant in the El-Minya location compared to the Sohag area, by 15% and 23% under normal conditions and 27% and 36% under heat stress, respectively. Increased seed and straw yields/plant under the normal conditions by 19% and 13% in the Sohag location and by 7% and 8% in the El-Minya location, respectively, compared with those under the heat stress conditions. These results are due to the lower temperature at the El-Minya location compared with that at the Sohag location. Depending on the results of each trait, genotypes responded differently in each situation. The traits analyzed across all genotypes are significantly influenced by years and geographical conditions. Environmental factors are the primary determinants of faba bean yield and yield components, according to several previously published studies [35, 39, 43, 44].

**Table 6:** The primary effects of years, locations, and faba bean genotypes on the investigated traits under normal (N) and heat stress (S) conditions

Factors	DSF		DSM		PH		NB/*P*		NP/*P*	
	*N*	S	*N*	S	*N*	S	*N*	S	*N*	S
Years										
2023/2024	95.82	92.66	322.02	302.06	215.64	204.52	8.13	6.96	31.12	25.84
2024/2025	48.20	45.03	160.70	149.24	105.22	99.38	4.13	3.56	14.95	12.19
LSD	0.23	0.36	NS	0.59	1.15	1.07	0.17	0.14	0.69	NS
Locations										
Sohag	56.34	51.67	171.49	150.09	107.70	97.60	4.29	3.51	11.70	8.07
El-Minya	39.48	40.98	150.15	151.55	107.13	106.17	3.72	3.36	18.93	17.27
LSD	0.23	0.36	0.53	0.59	NS	1.07	0.17	0.14	0.69	0.70
Genotypes										
Giza 843	48.13	46.80	159.72	148.93	110.36	106.41	3.81	3.36	16.27	13.55
Giza 716	49.32	48.82	165.32	153.25	107.70	99.35	3.86	3.23	15.00	11.26
Giza 429	46.36	45.38	159.47	148.84	111.99	108.16	4.37	3.61	15.00	11.78
Wadi 1	46.50	45.94	159.28	148.65	109.50	98.89	4.24	3.61	15.17	12.93
Sakha 1	50.11	46.51	164.97	154.30	110.51	102.62	4.52	3.39	15.46	13.86
Nubaria 3	46.06	45.02	157.91	150.06	105.62	103.81	3.74	3.23	18.47	14.31
Nubaria 1	49.81	47.28	166.13	152.75	100.41	96.89	4.70	4.26	12.56	10.03
Misr 3	47.58	45.51	157.08	151.00	105.46	100.36	3.28	2.97	15.49	12.41
Mariot 2	47.30	45.70	157.51	149.62	105.17	100.45	3.53	3.25	14.43	13.93
LSD	0.49	0.76	1.12	1.25	2.45	2.28	0.35	0.29	1.45	1.49
Factors	100-SW		SY/P		S/P		BY/P		HI	
	N	S	N	S	N	S	N	S	N	S
Years										
2023/2024	153.83	154.41	60.08	47.23	109.57	89.99	169.39	136.98	74.75	70.97
2024/2025	75.19	73.56	26.98	22.15	43.65	43.37	70.63	65.52	38.67	34.51
LSD	0.98	0.80	1.06	0.99	2.14	1.79	2.84	2.44	0.77	0.78
Locations										
Sohag	85.44	80.95	25.20	17.08	36.77	28.47	61.97	45.55	40.95	37.64
El-Minya	67.69	72.89	34.13	29.45	71.29	60.26	105.42	89.70	33.26	32.77
LSD	0.98	0.80	1.06	0.99	2.14	1.79	2.84	2.44	0.77	0.78
Genotypes										
Giza 843	76.20	78.01	31.96	26.39	48.09	48.98	80.05	75.37	40.52	36.26
Giza 716	76.82	77.02	29.12	20.43	50.07	40.52	79.18	60.95	37.79	33.95
Giza 429	74.50	76.59	33.92	21.52	56.36	48.34	90.27	69.87	38.60	32.07
Wadi 1	78.34	76.46	29.19	19.63	55.77	38.90	84.96	58.52	36.69	34.44
Sakha 1	75.12	77.70	29.42	24.14	58.78	47.09	88.20	71.23	35.41	34.98
Nubaria 3	78.49	77.40	32.91	24.54	57.15	45.16	90.05	69.70	38.13	36.16
Nubaria 1	80.02	79.75	24.26	24.05	54.34	42.90	78.39	67.16	32.56	36.38
Misr 3	75.29	76.56	29.42	24.09	53.88	42.79	83.30	66.88	37.20	35.91
Mariot 2	74.31	72.80	27.00	24.40	51.82	44.58	78.82	68.98	37.05	36.71
LSD	2.08	1.70	2.26	2.10	4.53	3.80	6.03	5.17	1.63	1.66

The traits key names can be found in Table 5

Under normal conditions, all genotypes recorded higher values for all evaluated traits than under heat stress conditions. The Nubaria 3 genotype under normal and stress conditions showed the lowest values for 50% flowering (46.06 and 45.02 days, respectively) and the highest values for the number of pods/plant (18.47 and 14.31, respectively). While the maximum values were registered by the Giza 429 genotype for plant height (111.99 and 108.16 cm) and by the Nubaria 1 genotype for number of branches/plant (4.70 and 4.26) and for 100 seeds weight (80.02 and 79.85 g) under normal and stress conditions, respectively, and for seed yield/ plant (33.92 g) and biological yield/plant (90.27 g) under normal conditions. As for 90% maturity, the Misr 3 and Wadi 1 genotypes had the highest values, at 157.08 and 148.65 days under normal and stress conditions, respectively. The Giza 843 genotype had the highest values for grain yield/plant (26.39 g), straw yield/plant (48.98 g) and for biological yield/plant (75.37 g) under stress conditions, and the highest harvest index (40.52%) under normal conditions. Finally, Sakha 1 and Mariot 2 genotypes had the highest straw yield/plant and harvest index traits, with values of 58.78 g and 36.71% under the normal and stress conditions, respectively. According to Janusauskaite and Razbadauskiene [45], certain faba bean cultivars exhibited uneven productivity responses to the weather during the growing season.

The results of this study indicated that heat-stress significantly reduced yield and other investigated traits compared with normal conditions. For example, the reduction in seed yield/plant ranged from 0.4% (Nubaria 1) to 22.4% (Giza 429). Our field tests’ findings supported previous research showing that heat stress has a detrimental impact on faba bean yield and yield components [2, 5, 7, 46]. These results demonstrated that some genotypes were more adversely affected by heat-stress, while other performed exceptionally well, as evidenced by their ability to yield even under such conditions. Therefore, a lower yield reduction, which is thought to be an important predictor of crop stability, indicates a cultivar’s capacity to function effectively under stress. Generally, Nubaria 3, Giza 843, and Giza 429 genotypes exhibited the ability to shorten the days to 50% flowering and 90% maturity, as well as to increase seed yield/plant and most of the studied traits under heat stress conditions. Therefore, they might be used as a source of short-duration cultivars to enhance the seed and straw yields in the Egyptian faba bean breeding program. It is more effective to select using yield and its constituent parts rather than just yield. The possibility of selecting an individual that deviates genetically from the mean of a segregating population clearly piques the interest of plant breeders [9].

Plant growth and production are ultimately harmed by high temperatures, which significantly affect plant physiobiochemical and anatomical parameters [47]. According to Yadav et al. [48], a shift in the temperature regime outside the ideal range during plant growth negatively affects phenophase and, ultimately, the crop yield due to altered physiological processes. Heat stress harms faba beans during flowering, when pollen viability is essential for successful reproduction. Reductions were likely due to gametophyte injury and subsequent fertilization failure [46]. Flower initiation, pollen viability, stigma receptivity, ovule viability, fertilization, seed set, grain filling, and seed quality were all significantly impacted by high temperatures. In the end, this results in low seed laying and extensive flower falling [3]. Poor seed production and low fodder yield are ultimately caused by high temperatures, which shorten crop growth periods and reduce the time available for biomass accumulation [3, 48]. Later stages of high-temperature stress can negatively affect respiration, photosynthesis, water relations, membrane stability, and hormone and primary and secondary metabolite levels. Moreover, key plant responses to heat stress include increased expression of certain heat shock proteins and other stress-related proteins, as well as the generation of reactive oxygen species (ROS) during plant ontogeny [49]. Plants use a variety of strategies to cope with heat stress, including maintaining membrane stability, scavenging ROS, producing antioxidants, accumulating and adjusting compatible solutes, inducing cascades of mitogen-activated protein kinase and Ca-dependent protein kinase, and, most notably, chaperone signaling and transcriptional activation [48]. Nubaria 3, Giza 843, and Giza 429 genotypes may be able to withstand heat stress thanks to these molecularly controlled pathways.

### Stress tolerance indices

The combination of heat tolerance indices across two years and two locations may provide a more useful criterion for evaluating the heat tolerance of the nine genotypes studied based on faba bean seed yield/plant (Table 7). Over two years and two locations, the seed yield/plant of nine faba bean genotypes under normal conditions (Yp) increased by roughly 12.2% compared with yields under heat-stress conditions (Ys). Fernandez [17] asserts that the best indices are those with a high correlation with seed yield under Yp and Ys conditions, and that the best measure for selection under stress conditions could distinguish genotypes with desirable, comparable yield from other groups. A more practical criterion for enhancing faba bean tolerance to heat stress would be selection based on a combination of indicators. A genotype tolerant of heat stress was identified by the highest heat tolerance indices for Yp, Ys, MP, GMP, STI, YI, YSI, DI, HM, and GOL and the lowest for SSI, TOL, YR, ATI, and SSPI heat tolerance indices.

**Table 7:** Comparison of stress indices for nine faba bean cultivars based on seed yield/plant under normal (Yp) and heat stress (Ys) conditions (averaged over two years and two locations)

Genotypes	Stress Tolerance Indices														
	Yp	Ys	SSI	TOL	MP	GMP	STI	YI	YSI	SI	YR	ATI	SSPI	HM	GOL
Giza 843	31.96	26.39	0.80	5.57	29.18	29.04	0.96	1.14	0.83	0.94	0.17	25.10	9.38	28.91	10.48
Giza 716	29.12	20.43	1.37	8.69	24.78	24.39	0.67	0.88	0.70	0.62	0.30	32.88	14.64	24.01	5.70
Giza 429	33.92	21.52	1.68	12.40	27.72	27.02	0.83	0.93	0.63	0.59	0.37	51.98	20.88	26.33	4.47
Wadi 1	29.19	19.63	1.51	9.56	24.41	23.94	0.65	0.84	0.67	0.57	0.33	35.50	16.10	23.47	5.11
Sakha 1	29.42	24.14	0.83	5.28	26.78	26.65	0.81	1.04	0.82	0.85	0.18	21.83	8.89	26.52	10.14
Nubaria 3	32.91	24.54	1.17	8.37	28.73	28.42	0.92	1.06	0.75	0.79	0.25	36.90	14.10	28.12	6.86
Nubaria 1	24.26	24.05	0.04	0.21	24.16	24.15	0.66	1.03	0.99	1.03	0.01	0.79	0.35	24.15	230.05
Misr 3	29.42	24.09	0.83	5.33	26.76	26.62	0.80	1.04	0.82	0.85	0.18	22.01	8.98	26.49	10.04
Mariot 2	27.00	24.40	0.44	2.60	25.70	25.67	0.75	1.05	0.90	0.95	0.10	10.35	4.38	25.63	19.77
Mean	29.69	23.24	0.96	6.45	26.47	26.21	0.78	1.00	0.79	0.80	0.21	26.37	10.86	25.96	33.62

The heat tolerance indices key names can be found in Table 4

Among faba bean genotypes, Giza 429 in the Yp condition, Nubaria 3 and Giza 843 genotypes in both conditions, had high seed yield; Sakha 1 and Misr 3 had intermediate seed yield in both conditions. Under Yp and Ys, the two genotypes Giza 843 and Nubaria 3 generally yielded the highest number of seeds. The Giza 843 genotype recorded the highest values for MP, GMP, and STI indices, followed by Nubaria 3 and Giza 429. These genotypes were shown to be the most heat-tolerant and desirable in both conditions. While Nubaria 1 and Mariot 2 genotypes had the lowest values for SSI, TOL, YR, ATI, and SSPI indices, and the highest values for YSI, SI, and GOL indices. As a result, these genotypes were identified as the most attractive and heat-tolerant under Ys. These indices appear to have been successful at selecting genotypes with modest seed yield under Ys, but they were unable to select genotypes with appropriate yield in either environments. As for YI and HM, the highest values were recorded for Giza 843 and Nubaria 3 genotypes, which are the most heat-tolerant and desirable under both conditions. On the other hand, according to most stress tolerance indices, the genotypes Wadi 1 and Giza 716 were identified as heat-prone. Except for the previous genotypes (sensitive and tolerant), all heat tolerance indices in this study categorized the other genotypes as either semi-tolerant or semi-sensitive to heat stress. Therefore, high rates of the MP, GMP, STI, YI, and HM indices should serve as the basis for selection under heat-stress conditions. Our results for stress tolerance indices are consistent with those of Memari et al. [21] and Rabie et al. [22] for faba bean under drought stress.

In Table 8, the genotype ranks for GMP and STI, as well as SSI and GOL, were the same. Additionally, very similar genotype ranks were observed among the SSI, GOL, YSI, SI, YR, ATI, and SSPI indices; between the GMP, STI, GM, and HM indices; and between the YS and YI indices, indicating that these parameters are equal for genotype selection. The estimates of heat tolerance indicators showed that it was inconsistent to identify heat-tolerant genotypes using a single criterion. Different genotypes were identified as heat-tolerant by various metrics. For instance, MP found that the Nubaria 1 genotype was heat-sensitive, whereas SSI found it heat-tolerant. The mean rank and standard deviation of ranks for all heat tolerance criteria were computed to identify the most suitable heat-tolerant genotypes across all stress indices. The genotypes Giza 843, Nubaria 3, Sakha 1, and Nubaria 1 had the best rank mean, with a low standard deviation and a rank sum of rank, according to the rank technique and all heat tolerance parameters. As a result, these genotypes were shown to be the most tolerant to heat stress. Additionally, the genotypes Giza 716 and Wadi 1 were the most heat-stress-sensitive, while other genotypes were either semi-sensitive or semi-tolerant. Similar findings indicate that, faba bean genotypes for MP, HM, GMP, and STI had nearly comparable ranks [19]; they all contributed to yield under both conditions, albeit with different weights [5]. For two or more stress indices, several faba bean genotypes generally displayed comparable ranks [19].

**Table 8:** Ranking method of nine faba bean genotypes based on stress tolerance indices

Genotypes Indices	Giza 843	Giza 716	Giza 429	Wadi 1	Sakha 1	Nubaria 3	Nubaria 1	Misr 3	Mariot 2
Yp	3.00	7.00	1.00	6.00	4.00	2.00	9.00	4.00	8.00
Ys	1.00	8.00	7.00	9.00	4.00	2.00	6.00	5.00	3.00
SSI	3.00	7.00	9.00	8.00	4.00	6.00	1.00	5.00	2.00
TOL	5.00	7.00	9.00	8.00	3.00	6.00	1.00	4.00	2.00
MP	1.00	7.00	3.00	8.00	4.00	2.00	9.00	5.00	6.00
GMP	1.00	7.00	3.00	9.00	4.00	2.00	8.00	5.00	6.00
STI	1.00	7.00	3.00	9.00	4.00	2.00	8.00	5.00	6.00
YI	1.00	8.00	7.00	9.00	4.00	2.00	6.00	4.00	3.00
YSI	3.00	7.00	9.00	8.00	4.00	6.00	1.00	4.00	2.00
SI	3.00	7.00	8.00	9.00	4.00	6.00	1.00	4.00	2.00
YR	3.00	7.00	9.00	8.00	4.00	5.00	1.00	4.00	2.00
ATI	5.00	6.00	9.00	7.00	3.00	8.00	1.00	4.00	2.00
SSPI	5.00	7.00	9.00	8.00	3.00	6.00	1.00	4.00	2.00
HM	1.00	8.00	5.00	9.00	3.00	2.00	7.00	4.00	6.00
GOL	3.00	7.00	9.00	8.00	4.00	6.00	1.00	5.00	2.00
Mean Rank	2.60	7.00	6.13	7.93	4.00	3.80	4.60	4.53	4.00
Variance	2.40	0.86	9.84	2.50	0.86	4.03	12.97	0.41	5.57
SDR	1.55	0.93	3.14	1.58	0.93	2.01	3.60	0.64	2.36
RS	4.15	7.93	9.27	9.51	4.93	5.81	8.20	5.17	6.36

*SDR* Standard deviation of ranks, *RS* Rank sum. The key names of the heat tolerance indices are listed in Table 4

**Fig. 1:**
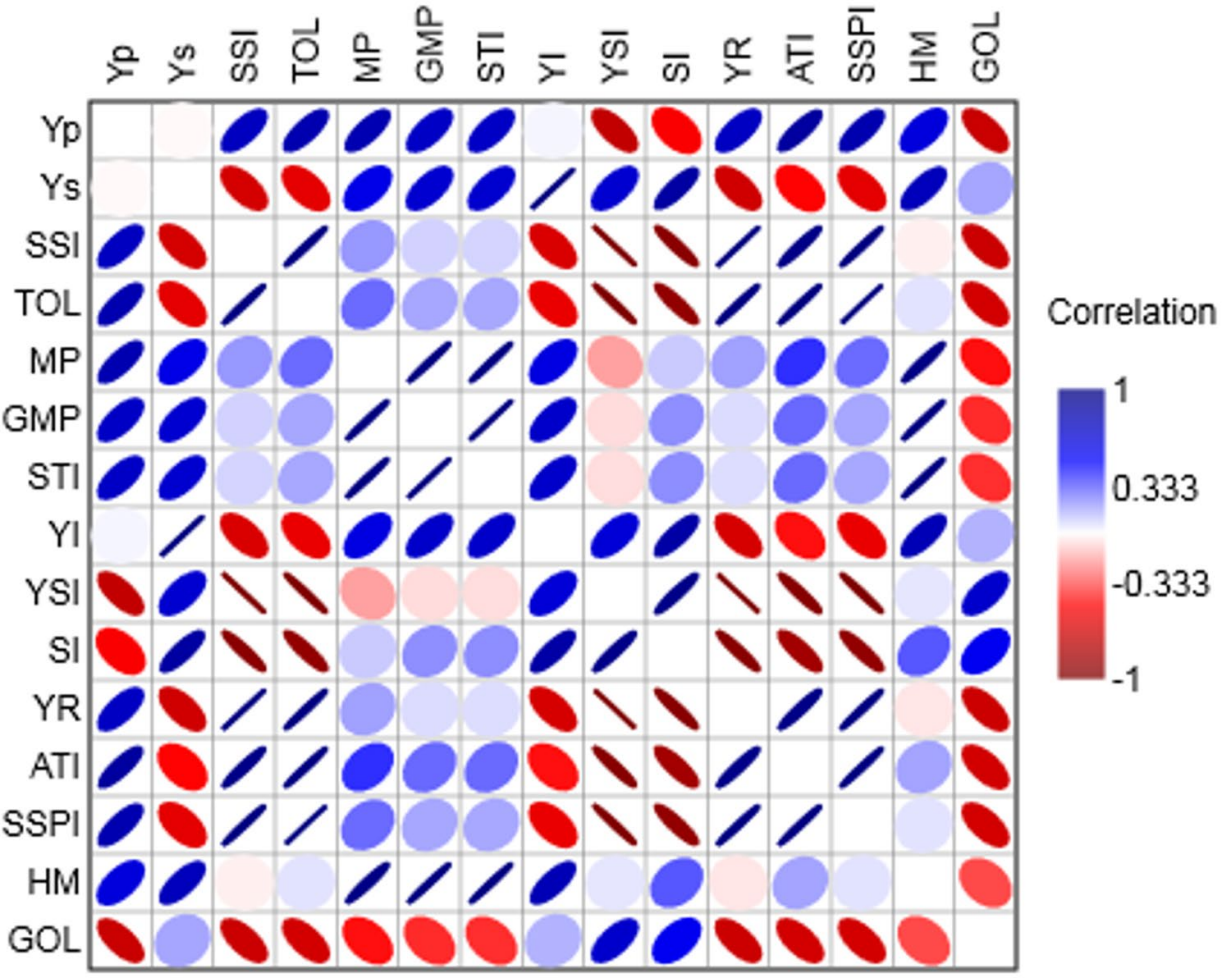
Correlation coefficient between Yp, Ys, and heat tolerance indices based on faba bean seed yield of studied genotypes. The key names of the heat tolerance indices are listed in Table 4

Pearson’s correlation analysis was used to examine the link between grain yield in both normal (Yp) and stress (Ys) conditions, and each of the heat stress indices for nine genotypes of faba bean (Fig. 1). High yield potential under normal growing conditions does not predict superior yield under stress conditions, as shown by the negative and non-correlation between Yp and Ys. For instance, the genotype Giza 429 yielded poorly under Ys but achieved the maximum yield under Yp. Therefore, it would be ineffective to use indirect selection for stress situations based on how well non-stress conditions performed. MP, GMP, STI, and HM indices significantly and positively correlated with seed yield in both Yp and Ys, suggesting that these indices were better at detecting high-yielding genotypes under both circumstances. We conclude that group A genotypes can be distinguished only under mild heat-stress using the MP, GMP, STI, and HM indicators. Additionally, Ys exhibited a significant and positive association with YSI (*P* < 0.05), YI, and SI (*P* < 0.01) indices, whereas Yp had a significant and positive association with SSI, TOL, YR, SSPI (*P* < 0.05), and ATI (*P* < 0.01). These connections showed that genotypes selected based on these indices were characterized by heat stress tolerance and would increase yield under stressful conditions. These associations were influenced by the severity of heat stress (the difference between Ys and Yp). In a similar vein, STI and YI are related, as both emphasize performance under stress conditions [5]. There was either no significant link or a substantial negative correlation between Ys and SSI, TOL, YR, ATI, and SSPI. Because yield decreased as indices increased under stress, these indices are useful for identifying faba bean genotypes with low yield and tolerance to heat stress. On the other hand, Yp was positively correlated with SSI, TOL, YR, ATI, and SSPI. Accordingly, selection based on SSI and TOL will increase yield under Yp, as indicated by the positive correlation between SSI, TOL, YR, ATI, and SSPI with Yp and the negative correlation between SSI, TOL, YR, ATI, and SSPI with Ys [21, 50]. Similar results have been reported in chickpea, where stress tolerance indices, particularly STI, YI, HM and GM, were identified as reliable criteria for selecting drought-tolerant genotypes with superior performance across contrasting environments. Furthermore, the authors highlighted that evaluating genotypes under both stress and non-stress conditions provides a more robust basis for identifying stable, high-yielding materials, as stress indices are strongly associated with yield performance and adaptive capacity under adverse environmental conditions [51]. Finally, Rizza et al. [52] demonstrated that the optimal genotypes could not be identified using a selection strategy based on the least yield drop under stress with relative to favorable conditions (TOL).

The highly significant positive correlation coefficients among SSI, TOL, YR, ATI, and SSPI indicate that they are strongly correlated in identifying heat-resistant genotypes. These findings suggested that these indices were equally capable of performing stress tolerance. The MP, GMP, STI, YI, and HM indices and the YI, YSI, and SI indices showed significant or highly significant associations. YSI had a substantial and positive correlation with both GOL (*P* < 0.05) and SI (*P* < 0.01). Except for YS with SSI, all assessed stress indices showed a substantial correlation with both grain yields (YP and YS). This outcome enabled the determination of whether the assessed stress indices were appropriate for identifying fababean genotypes as stress-sensitive or stress-tolerant [19]. Additionally, YSI, GMP, and MP parameters were linked to yield under stress, indicating that these constructs are appropriate for evaluating high-yield processing and stress tolerance [20].

**Fig. 2:**
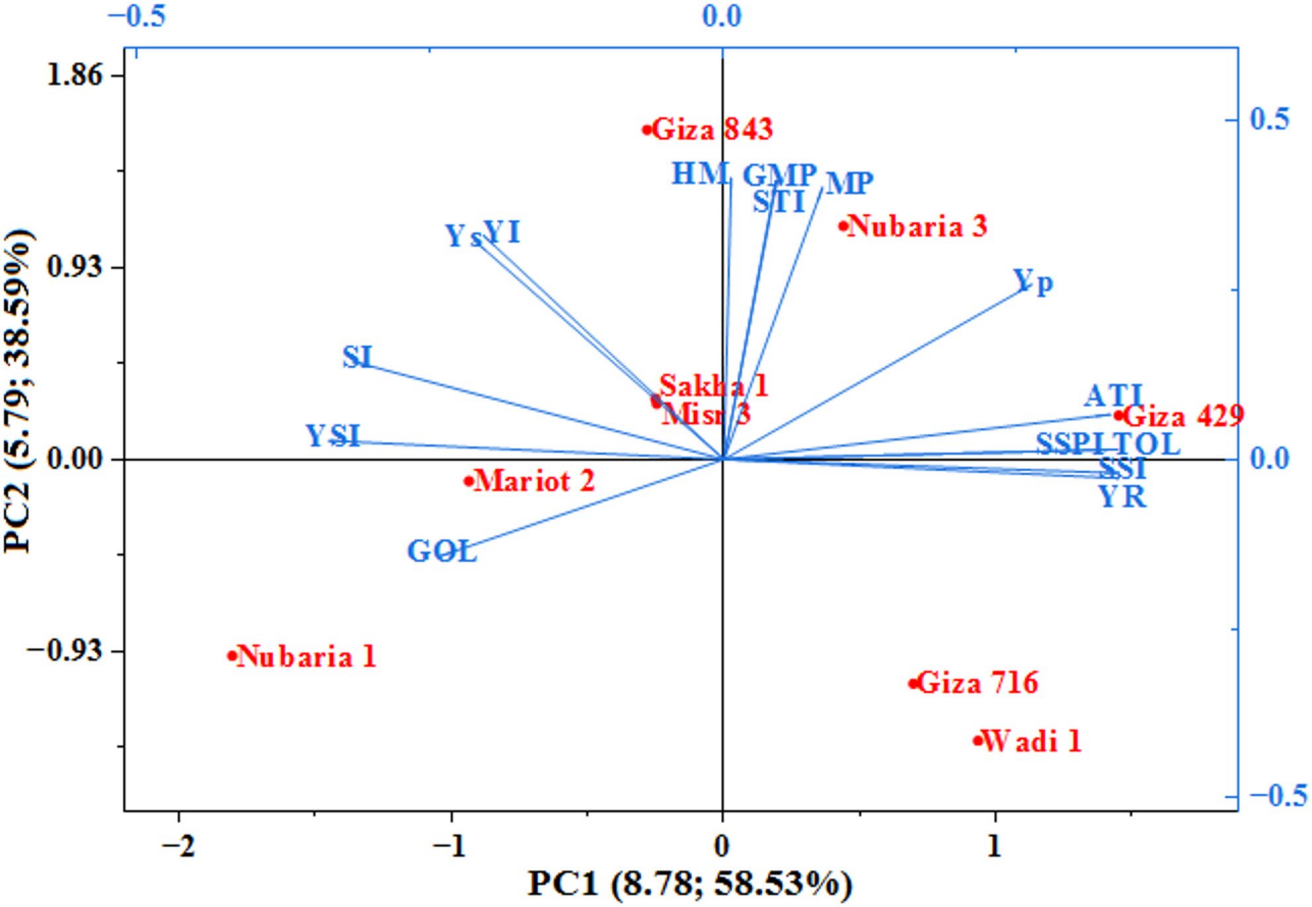
Biplot diagram based on PC1 and PC2 for heat stress indices and nine genotypes of faba bean for seed yield under normal (Yp) and heat (Ys) conditions. The key names of the heat tolerance indices are listed in Table 4

The association between heat tolerance indices based on seed yield for nine faba bean genotypes across two years and two locations was evaluated using principal component analysis (PCA). Seed yield (Yp and Ys) and heat indices have been reduced by PCA to just two components (PC1 and PC2), which can serve as the foundation for evaluating the correlation between heat tolerance indices (Fig. 2). Only PC1 and PC2, which account for 97.12% of the total variance of the variables, have eigenvalues greater than one, with values of 8.78 and 5.79, respectively. Thus, a biplot was created using PC1 and PC2. PC1 accounts for 58.53% of the total variance of the variables. Except for Ys, YI, YSI, SI, and GOL, it has a positive correlation with Yp and all stress indices across the genotypes of Giza 843, Giza 716, Giza 429, Wadi 1, and Nubaria 3. Across all genotypes (except Giza 716, Wadi 1, Nubaria 1, and Mariot 2), PC2 was positively associated with indices of Yp, Ys, and all stress indices except SSI, YR, and GOL, accounting for 38.59% of the total variance. Thus, the seed yield potential and heat tolerance can be referred to as PC1 and PC2, and selecting genotypes with high PC1 and PC2 is suitable for both Yp and Ys. With high PC1 and PC2 values under both Yp and Ys, the genotypes Giza 843, Nubaria 3, and Giza 429 are therefore superior genotypes and heat-tolerant.

Biplot analysis revealed near-zero angles, which indicate perfect positive correlations between YS and YI, between SSI and YR, between TOL and SSPI, and between STI and GMP, indicating that their genotype rankings are identical. The acute angle (below 90 degrees) and the obtuse angle (above 90 degrees) between variables in a biplot represent positive and negative correlations, respectively. The PCA and Pearson’s correlation analysis findings are comparable. PCA reveals a highly positive association, indicating that the genotypes in these indices are ranked similarly. This implies that selection based on these indices will increase seed yield in both circumstances. Since the genotypes Giza 843 and Nubaria 3 were found to be associated with Yp, Ys, and the MP, GMP, STI, YI, and HM indices, these indices under Yp and Ys are the most suitable indices for selecting genotypes. In stressful conditions, superior genotypes can be identified using YSI, GMP, and MP indicators [20]. According to the PCA analysis, genotypes with varying degrees of stability and stress tolerance may be distinguished using STI and YSI, respectively [53]. The PCA results validates the correlation analysis and provide useful information from the data. The genotypes combine high yield potential under both stress and non-stress conditions with environmental stability, as indicated by stress tolerance indices [5, 19].

**Fig. 3:**
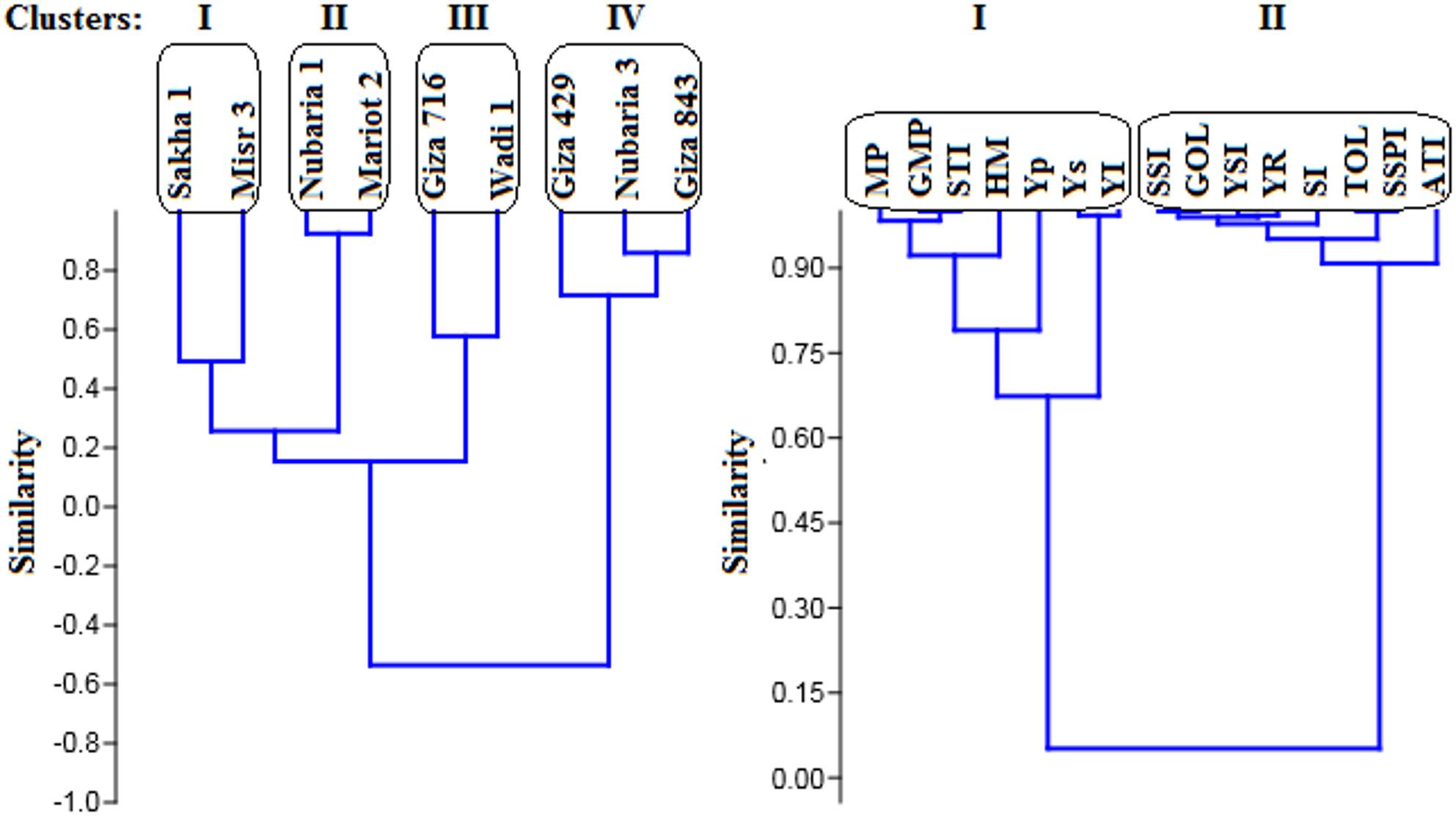
Cluster analysis for nine faba bean genotypes as well as Yp, Ys and heat tolerance indices. The key names of the heat tolerance indices are listed in Table 4

Cluster analysis was used to identify genetic variance, genotype proximity, and similarity among stress indices (Fig. 3). The genotypes were divided into four clusters based on Yp, Ys, and other stress indices. The genotypes in each cluster were quite similar. As a result, there was significant variation among faba bean genotypes that were examined for heat tolerance. It is anticipated that greater heterosis and more robust plants will result from hybridization or crossover between distantly related populations. There were two genotypes in each of the first (I), second (II), and third (III) clusters: Sakha 1 and Misr2, Nubaria 1 and Mariot 2, and Giza 716 and Wadi 1, respectively. The genotypes Giza 429, Nubaria 3, and Giza 843 made up the fourth cluster (IV). Because the genotypes in the IV cluster had high MP, GMP, STI, YI, and HM index values, they were regarded as the most desirable genotypes under Yp and Ys (tolerant group). However, the genotypes in cluster I are vulnerable to heat stress, whereas those in clusters II and III are semi-tolerant. Cluster analysis revealed that genotypes in cluster IV were superior for the MP, GMP, STI, YI, and HM indices; therefore, it is advised to cultivate these genotypes under stress. These findings concur with those of Balko et al. [4].

Based on the genotypes under normal and stress conditions, the cluster analysis for Yp, Ys, and other stress indices tended to group into clusters (I and II). Yp, Ys, MP, GMP, STI, YI, and HM indices made up cluster (I) The other stress indices under study were included in Cluster (II) The tree diagram identified the maximum similarity between the indices within each group. Nonetheless, the two groups’ indices showed the greatest disparity. These findings showed that heat tolerance indices were very comparable within each cluster. However, the findings showed that the two clusters differed. The evaluated genotypes were divided into sensitive and tolerant groups based on heat tolerance indices; hierarchical clustering identified four groups [37]. Additionally, Chetto et al. [35] and Elshafei et al. [54] found that the examined genotypes clustered into two and five major clusters, respectively, with significant similarity indices. Tadele and Shiferaw [19] concluded that stress-tolerant genotype screening was equally dependent on characteristics that identified genotypes in the same order.

### Principal component analysis (PCA)

**Fig. 4:**
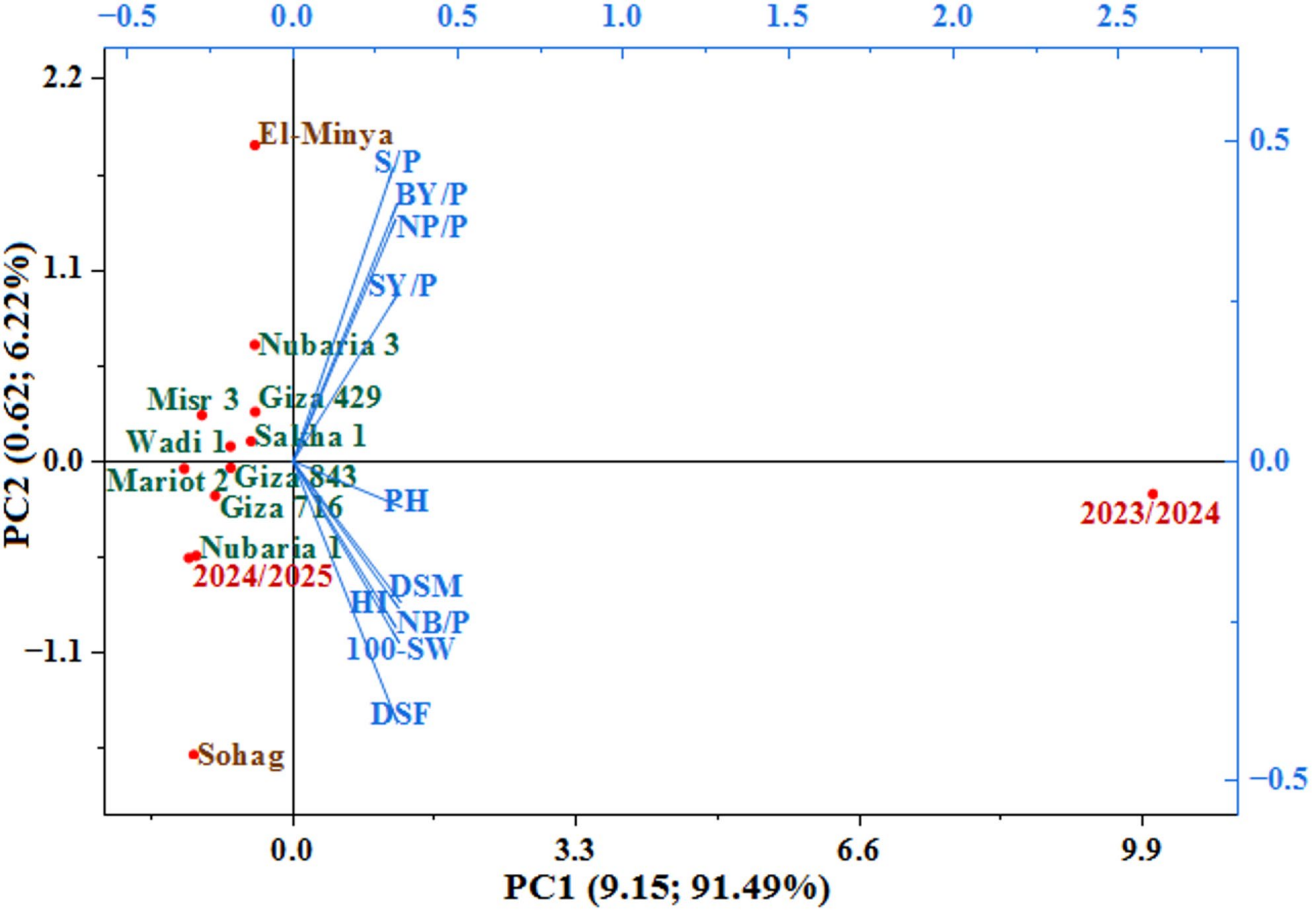
PCA for seed yield and other studied traits of nine faba bean genotypes in two years and locations across normal conditions. The traits key names can be found in Table 5

**Fig. 5:**
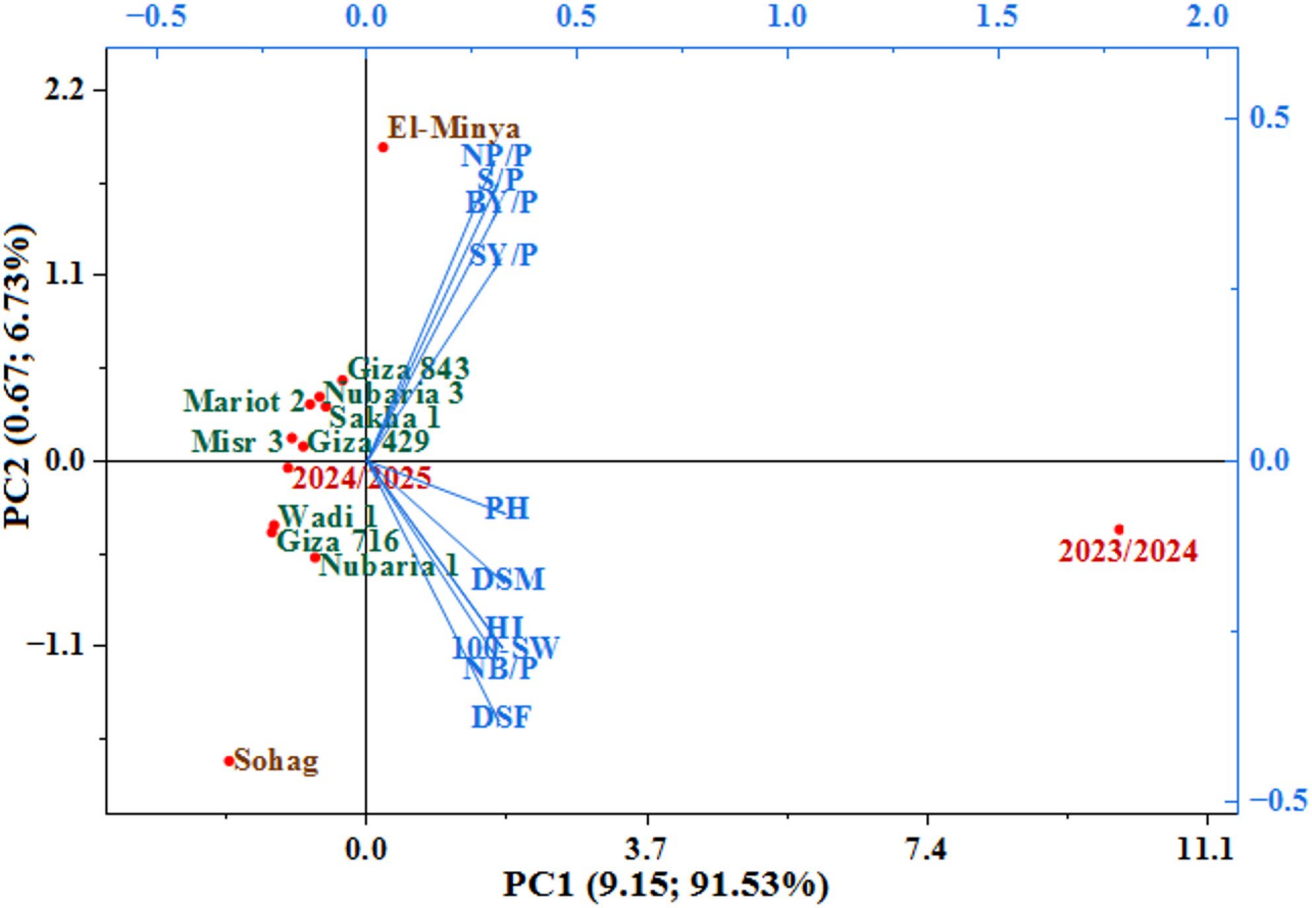
PCA for seed yield and other studied traits of nine faba bean genotypes in two years and locations across heat stress conditions. The traits key names can be found in Table 5

PCA analysis was then conducted out to assess the interaction between genotype and environments (years and location) under normal and heat stress conditions. Figures 4 and 5 shows PC1 and PC2 for the experimental conditions and for every trait under normal and heat stress conditions, respectively. Although the number of PCs generated equaled to the number of traits examined in both conditions, the 10 PCs explained 100% of the overall variation among traits across 9 faba bean genotypes, 2 years, and 2 locations under study. Only PC1, with an eigenvalue > 1 accounted 91.49% and 91.53% of the total variation among the variables under investigation across normal and heat stress conditions, respectively, according to the PCA results. Under normal and heat-stress conditions, the remaining PCs accounted 8.51% and 8.47%, respectively, with eigenvalues smaller than 1. Similar results showed that the first two PCs accounted for 63.9% and 59.3% of the variance across control conditions and the stress treatment, respectively, consistent with the distribution of the genotypes and studied traits [4] of the variance across control conditions and the stress treatment, respectively, with the distribution of the genotypes and studied traits. Also, PC1 and PC2 explained for 83.64% and 16.36% [40] and 44.38% and 13.40% [35] of the total variance, respectively.

All traits under study showed genetic variability at the site impacts across growing years in both situations, as shown in the biplot. Therefore, the PC1 and PC2 results can be used to summarize the original variables in subsequent data analyses in both situations, as well as to explain the overall variance and the PC loadings. Additionally, PC1 captures a high percentage of variance in both conditions, indicating that it successfully differentiation faba bean genotypes based on how their performance across the variables under investigation in different environmental conditions.

The biplot of PC1 and PC2 shows that all studied traits were positively associated with the experimental factors in both conditions, with a steep angle between them. A strong positive correlation was found among SY/P, BY/P, NP/P, and SP traits, as well as among other studied traits in both conditions, reported earlier by El-Abssi et al. [37], Barilli et al. [5], and Sallam et al. [40]. These findings suggest that selecting for these traits could increase the productivity potential of faba beans. Plant height and yield component traits were positively correlated with seed yield [2, 12, 54, 55], whereas earliness was negatively correlated with seed yield [12]. Correlations analysis reveals statistically significant relationships among the various characteristics, providing important insights into the morphological and physiological tactics used by the genotypes under study [35]. Positive assocations across features for both normal and heat-stressed circumstances, according to PCA, show that additional quantitative traits can increase yield, enhancing overall performance [56].

Figures 4 and 5 show the distribution of genotypes, years, and locations along the ordinate axes for both conditions based on PC1 and PC2. PC1 showed a positive correlation with every trait under investigation, the growing year 2023/024 under both conditions, and El-Minya locations under heat stress (the first and fourth quarters). These results suggest that they can be simultaneously selected to shape early developing, high-yielding genotypes in both situations and that they significantly contribute to the phenotypic variability of faba bean genetic resources. PC1 separates genotypes by agronomic performance and is associated with the performance of the assessed faba bean genotypes [37]. In contrast, PC2 showed positive correlations with NP/P, SY/P, S/P, and BY/P traits with the El-Minya location under both conditions; with genotypes of Giza 429, Wadi 1, Sakha 1, Nubaria 3, and Misr 3 under normal conditions; and with Giza 843, Giza 429, Sakha 1, Nubaria 3, Misr 3, and Mariot 2 under stress (the second quarter). Elite lines’ performance was differentiated by PCA between the first and second growing seasons, with PC1 accounting for most of the differences [35]. Genotypes linked to higher seed yield and related traits were successfully distinguished from those linked to lower grain yield and related traits by the PCs biplot. According to El-Abssi et al. [37], the genotypes exhibited a variety of multidimensional spaces and plots with varying bottom-to-top distances. Owing to their closeness, the El-Minya location’s genotypes Nubaria 3 in both conditions, Giza 429 under normal conditions, and Giza 843 under heat-stress conditions were significance for faba bean early maturity and production. Although direct selection is ineffective, PCA as described by PC1 and PC2 provided guidance for selecting genotypes and traits for early maturity and boosting faba bean productivity under both conditions in Egypt. Over two growing seasons, PCA revealed that elite lines responded differently to environmental conditions [35].

## Conclusions

The low-yielding, stress-tolerant nature of faba bean is well-known. The development of heat-tolerant genotypes would benefit from research on high-temperature tolerance or susceptibility and its relationship to seed yield and other associated traits in faba bean. This work serves as a proof of concept for the applicability of genotype evaluation in faba bean and testing under stress across time and geography. Nubaria 3, Giza 843, and Giza 429 genotypes were found to be superior based on the univariate and multivariate model results across years and location conditions under heat-stress condition. It also exhibited very low reductions in seed and straw yields under heat-stress conditions. It may be recommended for late sowing dates in Egypt. In the future, a diverse faba bean population that includes these genotypes, including those with heat tolerance, will be developed and used for further studies.

## Data Availability

The datasets generated during the current study are available from the corresponding author on reasonable request.
